# Design and implementation of an intelligent monitoring system for household added salt consumption in China based on a real-world study: a randomized controlled trial

**DOI:** 10.1186/s13063-020-04295-1

**Published:** 2020-04-21

**Authors:** Jinli Xian, Mao Zeng, Rui Zhu, Zhengjie Cai, Zumin Shi, Abu S. Abdullah, Yong Zhao

**Affiliations:** 1 School of Public Health and Management, Chongqing Medical University, Yixueyuan Road, Yuzhong District Chongqing, Chongqing, 400016 CN China; 2 Research Center for Medicine and Social Development, Chongqing Medical University, Chongqing, 400016 China; 3 The Innovation Center for Social Risk Governance in Health, Chongqing Medical University, Chongqing, 400016 China; 4 Human Nutrition Department, College of Health Sciences, QU Health, Qatar University, Doha, Qatar; 5 Global Health Program, Duke Kunshan University, Kunshan, 215347 Jiangsu Province China; 6 Duke Global Health Institute, Duke University, Durham, NC 27710 USA; 7 School of Medicine, Department of General Internal Medicine, Boston University Medical Center, Boston, MA 02118 USA; 8 Chongqing Key Laboratory of Child Nutrition and Health, Children’s Hospital of Chongqing Medical University, Chongqing, 400014 China

**Keywords:** salt consumption, intelligent monitoring system, real-world study, household

## Abstract

**Background:**

A high intake of salt is a major risk factor for cardiovascular diseases. Despite decades of effort to reduce salt consumption, the salt intake in China is still considerably above the recommended level. Thus, this study aims to design and implement an intelligent household added salt monitoring system (SALTCHECKER) to monitor and control added salt consumption in Chinese households.

**Methods:**

A randomized controlled trial will be conducted among households to test the effect of a SALTCHECKER in Chongqing, China. The test modalities are the SALTCHECKER (with a smart salt checker and a salt-limiting WeChat mini programme) compared to a salt checker (with only a weighing function). The effectiveness of the system will be investigated by assessing the daily added salt intake of each household member and the salt consumption-related knowledge, attitude and practice (KAP) of the household’s main cook. Assessments will be performed at baseline and at 3 and 6 months.

**Discussion:**

This study will be the first to explore the effect of the household added salt monitoring system on the reduction in salt intake in households. If the intelligent monitoring system is found to be effective in limiting household added salt consumption, it could provide scientific evidence on reducing salt consumption and preventing salt-related chronic diseases.

**Trial registration:**

Chinese clinical trial registry (Primary registry in the World Health Organization registry network): ChiCTR1800018586. Date of registration: September 25, 2018.

## Background

Excessive dietary intake of sodium is associated with the increasing risk of hypertension and cardiovascular diseases [1]. The current average salt intake in Chinese adults (10.4 ± 0.2 g/d) is higher than the recommended level of the World Health Organization (5 g/d) and that of the Chinese Nutrition Society (6 g/d) [1–3]. In China, most dietary sodium intake is from salt added in home cooking [4, 5]. Therefore, innovative interventions to reduce salt intake at the household level should be a priority in China.

Several salt reduction programmes have been carried out around the world and are considered to be effective [6–9]. Programmes in developed countries have achieved excellent results; they promote food nutrition labels, educate the public, strengthen cooperation with food companies and reduce salt content in processed foods [10–13]. In China, evidence-based strategies to reduce salt intake are available [14–19]. The common salt reduction initiatives are salt substitutes, salt-restricting tools and health education [20]. However, the implementation of these strategies is limited [10, 14–19], mostly due to the high price of salt substitutes [20], inappropriate belief in salt-using experience and lack of awareness about the harms of excessive salt intake in residents, and the complex operation of the salt-limiting spoon [21].

Increasing salt-related knowledge levels and favourable attitudes are beneficial to improve salt-limiting behaviours. The knowledge of salt use and its impact on health is limited among residents in China [22]. In an earlier study in China, favourable actions of dietary sodium reduction were more likely to occur among those who were aware of the link between sodium and hypertension, and less likely to occur among those who had unfavourable attitudes toward dietary sodium reduction [7]. Therefore, evaluating the salt consumption-related knowledge, attitude and practice (KAP) is essential to initiate salt-limiting nutritional education and promote healthy salt behaviour.

Currently, the main methods to monitor salt intake include 24-hour urine collection, spot urine collection and dietary surveys [23]. However, these methods have some shortcomings. Although, the 24-hour urine collection is reliable for evaluating salt intake [24], the laborious collection and detection of urine samples and the lack of a suitable method for correctly identifying incomplete samples were reported as potential barriers [25]. Spot urine sampling is a convenient and affordable alternative to estimate 24-hour urinary sodium excretion, but has the disadvantages of difficult selection of the time of spot urine sampling monitoring, and non-representative and unsuitable spot urine sampling for individual assessment [25, 26]. Dietary recalls and weighed diet records are time-consuming and labour-intensive and often underestimate actual salt intake [25]. Therefore, an accurate and convenient method to assess the salt intake of individuals is needed.

The Internet of Things (IOT) is the network of physical devices, vehicles, home appliances and other items embedded with electronics, software, sensors, actuators and connectivity, which enable these things to connect, collect and exchange data [27]. Currently, IOT technology is cumulatively applied in health education and promotion, such as physical activity recognition and monitoring [28], personal health records [29], smart care for specific populations [30–32] and risk prediction [33]. However, IOT technology has not yet been applied to monitor added salt consumption. WeChat, a social media platform, similar to Facebook, widely used in China [34], has great potential to be integrated with IOT technology for salt reduction intervention. A WeChat mini programme appears to be similar to general apps, however, there is no need to install or uninstall them on the smartphone; this feature improves accessibility and convenience [35]. This study will combine IOT technology, a WeChat mini programme and a salt-limitation intervention to provide an innovative idea and approach for the monitoring and reduction of household salt intake.

In recent years, studies in the real-world settings have become increasingly important in providing evidence of treatment effectiveness in clinical practice [36]. Real-world research is made necessary by the various factors that can play an important role in modulating effectiveness in real life [37]. Real-world evidence comes from the integration of data from various clinical practices and personal health management with features of integration, personalisation and authenticity [38]. In our study, the daily added salt intake of each household member and the salt consumption-related KAP of the household’s main cook will be collected, which will be used as real-world data from households.

## Methods/Design

### Aim

This study aims to (1) develop an intelligent added salt monitoring system based on real-world study and (2) evaluate the effectiveness of the system in reducing the daily added salt intake of each household member and improving the salt consumption-related KAP of the household’s main cook.

### Design

This pilot study comprises two stages. Each stage achieves one of the objectives described above. Additional file 1 provides the complete Standard Protocol Items: Recommendations for Standard Protocol Items: Recommendations for Interventional Trials (SPIRIT) checklist.

#### Stage 1: Development of intelligent added salt monitoring system (SALTCHECKER)

##### The Development of SALTCHECKER

**Fig. 1:**
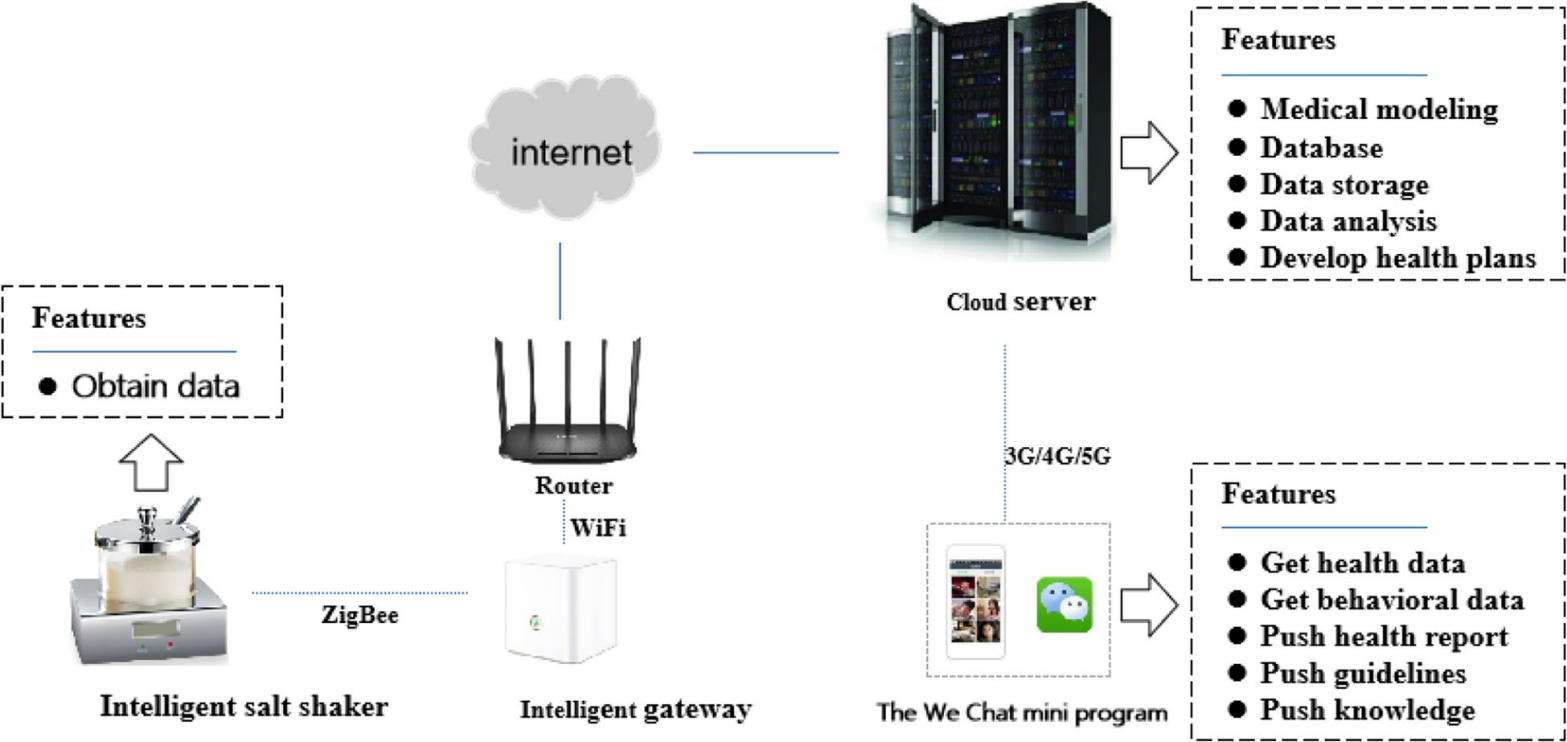
System functional structure diagram

The entire system will be divided into three parts: front-end sensing, cloud analysis and terminal interaction (Fig. 1).

The front-end sensing system is also called the “smart salt checker”. It has a salt container and an electronic salt sensor. The electronic salt sensor is equivalent to a self-designed electronic weighing scale on the base of the salt container, which can weigh the salt in the container, with a weighing range of 0–250 g and a sensitivity of 0.2 g. After the user takes the salt each time, and the container is placed on the base with the lid closed, the processing unit of the electronic salt sensor can immediately detect and record the weight and the time of salt used.

The cloud analysis system can automatically calculate the individual added salt intake of each household member according to the information acquired from the terminal interaction system. The daily added salt intake of each household member will be calculated on the basis of the distribution of staple food consumption of residents in the household. Daily intake estimates of added salt for each household member (g/standard) = (30 days’ intake of added salt by the surveyed household × standard daily number of each member in the household/standard daily number of all people in the household)/30 [39].

The terminal interaction system is a salt-limiting WeChat mini programme; through the programme, basic data can be acquired of the participating household’s main cook and other household members, such as their age, gender, occupation, marital status, education level, height, weight, labour intensity, health status, the number of people per meal and meal ratio per day for members aged>2 years old in their household. Participants with hypertension will also be encouraged to provide data on blood pressure, and the time and tool of blood pressure measurement.

Overall, the SALTCHECKER contains three key features: (1) self-monitoring: to automatically calculate the daily added salt intake of each household member, (2) questionnaires: to investigate the basic demographic information of the household’s main cook and other household members and salt consumption-related KAP of the household’s main cook, (3) personalised reports: through salt-limiting WeChat mini programme, professional nutrition experts in our research group will provide the daily added salt intake of each household member and corresponding healthy salt-using suggestions for specific household members to the household’s main cook.

##### Qualitative interview

During the SALTCHECKER development, a semi-structured in-depth interview will be conducted among the household’s main cook, information technology personnel and nutritionist to determine the content of the system. The selection of candidates for the interview will follow dynamic sampling and information saturation principles until no new useful information can be collected by adding new subjects. Each interview will take around 20 minutes. Interviews will be digitally audio-recorded after obtaining the informed consent of the interviewee. From the interview, the feelings and suggestions for the content of the personalised report, privacy and security protection; system professionalism and functional diversification; health guidance or management category; and other characteristics of the system can be identified. Then, the function of the system can be improved to meet the needs of the residents. Based on the qualitative interview and evidence-based literature, the SALTCHECKER will be finalised and the main content of the personalised report developed.

#### Stage 2: Evaluation of the effectiveness of SALTCHECKER

To evaluate the effectiveness of SALTCHECKER, we will (1) assess the time trend of salt intake change during the study period and (2) compare the individual salt intake of the household member and the salt consumption-related KAP of the household’s main cook between the intervention group and the control group at baseline, 3 and 6 months.

### Study sample

To calculate sample size, the following formula will be used:

$$ N=\frac{{\left({U}_{\alpha }+{U}_{\beta}\right)}^2\times 2P\left(1-P\right)}{{\left({P}_1-{P}_2\right)}^2} $$

where α = 0.05, β = 0.1, and then U_0.05_ = 1.96, U_0.1_ = 1.28. According to a previous study in China, the proportion of daily per capita salt consumption ≤6 g was 37.91% and the proportion increased to 45.91% after using salt-control spoons in participating households [16]. Thus, in this study, the increase in low salt consumption (≤6 g/d) with SALTCHECKER from about 38% (*P*_*1*_) to some 46% (*P*_*2*_), *P* = (*P*_1_ + *P*_2_)/2 = 0.42 was assumed. The sample size is calculated to be 799 cases. According to some similar salt-limiting trials, the dropout rate was 0.5–5% [18, 40–42]. And sampling error and invalid questionnaires may be 10–15%. Therefore, it was assumed 10–20% should be added on the basis of the estimated sample size, so 879–959 participants will be needed. The data of the tabulation on the 2010 population census of the People’s Republic of China (No. 1) indicated that the average household size in Chongqing is 2.70 (person/household) [43]. Therefore, 300 households’ main cooks in each of the intervention group and control group will be investigated in this study.

### Recruitment

The households will be recruited from Chongqing, China. The study will be advertised in cooperative communities. The following strategies will be used sequentially until the desired sample size is achieved: (1) posters and project leaflets will be distributed; (2) advertisements will be circulated on social media, such as WeChat and Weibo platforms; (3) the project will be presented in the community by project manager or project members in the form of health seminars; and (4) the use, function and advantages of the SALTCHECKER will be demonstrated on-site.

Inclusion criteria: (1) the household’s main cook (involved mainly in household cooking); (2) plan to be living in Chongqing, China for at least 6 months; (3) own a mobile phone (iOS or Android) with Internet access; (4) and agree to have the WeChat mini programme on their mobile phones.

Exclusion criteria: (1) currently using a self-monitoring system or device to track or log living behaviour in household members; (2) having been involved in other salt experiments in household members; and (3) prior history of cognitive or verbal impairments or other diseases that may impact on study participation.

Interested household main cooks or their household members who are already using a self-monitoring system or tracking device will be excluded to avoid the potential confounding effect. The reason is that popular health apps or tracking device frequently implements various behavioural change strategies, which may affect participants’ behaviour.

On the basis of a computer-generated list in the statistical software IBM SPSS version 22.0 (IBM Corp, Armonk, NY, USA), we will randomly select 600 households’ main cooks (who meet the above-mentioned inclusion criteria) from the available household main cooks in the project database. Written informed consent will be collected from all the participants.

### Procedure

**Fig. 2:**
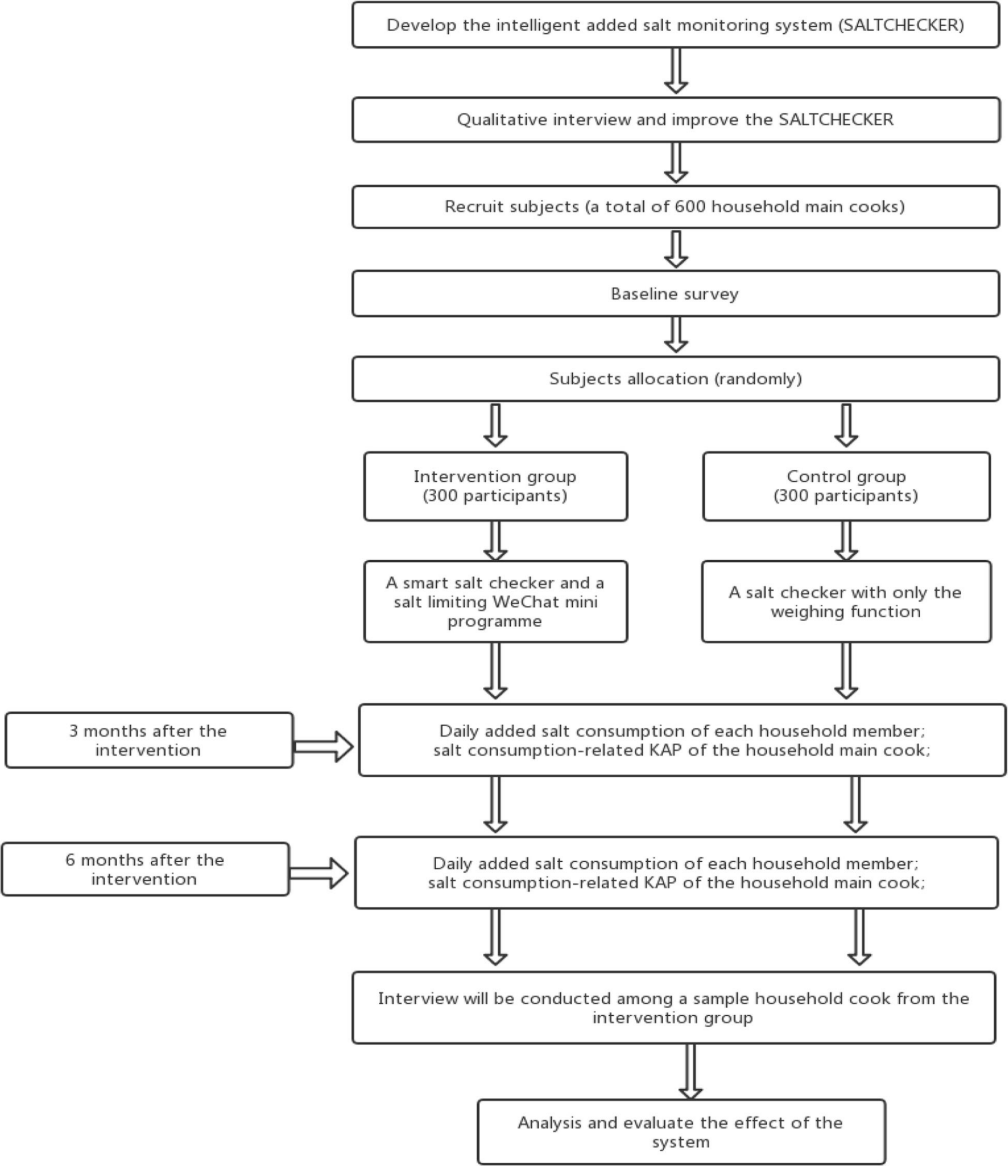
Technology roadmap for this project

A baseline survey will obtain the household demographic information and the salt consumption-related KAP of the household’s main cook. In the test period, the household’s main cook in the intervention group will use the smart salt checker and the salt-limiting WeChat mini programme, while the household’s main cook in the control group will use the smart salt checker with only the weighing function. The data of the daily added salt intake of each household member will be automatically calculated using SALTCHECKER. And the salt consumption-related KAP of the household’s main cook will be assessed at the beginning of the test (i.e. at baseline), the third month and the sixth month after the test. Finally, the effect of the household added salt monitoring system will be evaluated by changes in daily added salt intake of each household member and the salt consumption-related KAP of the household’s main cook. The technological roadmap is shown in Fig. 2.

### Baseline survey

#### Daily added salt intake of each household member

The front-end sensing system in the SALTCHECKER will automatically obtain the data of the added salt consumption of participating households, and the data will be collected and stored every day. The daily added salt intake of each household member will be calculated based on a formula, described in the Development of SALTCHECKER stage 1 section.

#### Questionnaire survey

A self-administered questionnaire will be designed based on the local and international literature to assess the salt consumption-related KAP of the household’s main cook. The questionnaire will be sent to the household’s main cook through the salt-limiting WeChat mini programme. After the pre-investigation, the reliability and validity of the questionnaire will be tested. The questionnaire will include the following three parts:

- Knowledge of salt consumption, such as the daily recommended intake of salt, high-salt diet hazards, recognition of seasonings and food with high sodium, etc. The knowledge of salt consumption is measured (0 = no, 1 = yes, 0 = do not know), and some items require reverse coding.
- The attitude of salt consumption, such as the attitude to salt-limiting or low-salt diets, awareness and intention of choosing a low-salt diet, etc. will be assessed using the 5-point Likert scale (1 = strongly disagree to 5 = strongly agree) questions.
- The practice of salt consumption, such as the use of low-sodium salt, proper use of salt-limiting spoons, selection of low-salt food, requiring low-salt food in restaurants, selection of food based on the nutrient list, etc. will be measured using the 5-point Likert scale (1 = strongly disagree to 5 = strongly agree) questions.

A total score will be calculated for the above items, and a high score will represent a high KAP.

#### Randomization and blinding

After the baseline survey, 600 participating households’ main cooks will be randomly divided following a 1:1 ratio into the intervention group and the control group by the household demographic information, with 300 households in each group. The study coordinator will be responsible for the randomization and verification of the computer-generated intervention assignment with a randomization table. All the investigators, except for the study coordinator, will be blinded. The participants will be blinded and instructed not to inform the other participants of the treatment they receive.

### Intervention

#### Intervention group

In the intervention period, the participating household’s main cook in the intervention group will install the smart salt checker in their households, and receive the personalised reports and health education through the salt-limiting WeChat mini programme monthly. Personalised reports will mainly contain the daily added salt intake of each household member, the adverse impact of excessive salt consumption and the salt-limiting suggestions. The health education includes salt-limiting guidance and targeted health articles. The salt-limiting guidance will be provided by professional nutrition experts through the salt-limiting WeChat mini programme. People from the target population can select the intervention content depending on their specific situations and interests. The participating household’s main cook can propose questions and ask for suggestions from the nutrition experts in real time through the WeChat mini programme. The intervention period will last for 6 months. Through the personalised reports and health education, we seek to provide the participating household’s main cook with useful and effective guidance on two aspects:

1. Provide the daily added salt intake of each household member.
2. Convey knowledge and suggestions on the negative effects of excessive salt consumption and ways to limit salt consumption and maintain healthy behaviour.

#### Control group

The household’s main cook in the control group will have access to the smart salt checker with only the weighing function.

#### Qualitative interview of participating household cooks

**Fig. 3:**
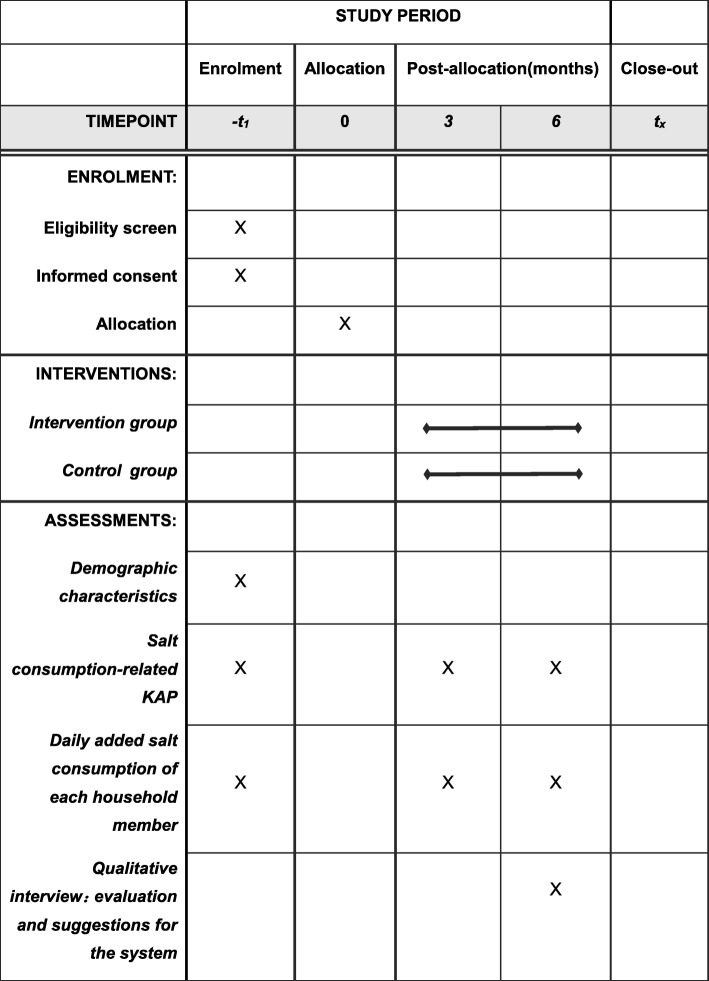
Schedule of surveys

After 6 months of intervention, a semi-structured in-depth interview (20–30 minutes each) will be conducted among a sample household main cooks from the intervention group. The selection of subjects for the interview will follow the dynamic sampling and information saturation principles until no new useful information can be collected by adding new subjects. Several open-ended questions will measure the household’s main cook’s satisfaction of the system (i.e. ‘Would you like to recommend the system to friends who also want to reduce their salt consumption?’), the effect of the system on salt reduction (i.e. ‘What are the benefits of using the system?’), suggestions on optimisation and intelligence of interface (i.e. ‘What are the limitations of using the system?’) and other aspects to conduct a primary evaluation of the monitoring system. A sample of those who do not continue using the system will be interviewed and the reasons documented. Figure 3 shows the schedule of enrolment, assessment and intervention.

#### Primary outcomes

1. Daily added salt intake of each household member, assessed at baseline, 3 and 6 months.
2. Salt consumption-related KAP of the household’s main cook, assessed at baseline, 3 and 6 months.

### Quality control

#### Online questionnaire survey

The participating household’s main cook can log on to the WeChat mini programme with the password set by themselves to complete the online questionnaires. The system will automatically send the electronic KAP questionnaire to the participants at baseline, 3 and 6 months. If the participant does not complete the questionnaire within 1 week, a text message will be sent or call made to remind him or her. A branched algorithmic structure will be used to enhance the confidentiality and accuracy of responses and reduce the risk of exposing sensitive information.

#### Trial operation and maintenance of the system

Before the start of the study, a test operation of the system will be carried out in the participating households by the developer of the system. During the study, detailed calibration and inspection of the system will be regularly conducted to ensure the accuracy and reliability of the intervention and measuring results.

#### Data of the daily added salt intake integrity

Before the study, the participants will be informed that they will be provided with salt for free, which aims to make them use the added salt only from the smart salt checker container in the survey period. And the measured outliers will be excluded, which may be caused by using too much salt for preserving food, adding too much salt to the smart salt checker or other reasons. A certain reward will be given to the households’ main cooks who actively participate in the programme every month.

### Analysis strategy

Statistical analyses will be conducted using SPSS version 22.0 (IBM Corp, Armonk, NY, USA). Descriptive statistics will be calculated for all the variables under examination. The counting data will be described by frequency and percentile, and the metrological data description will use mean, standard deviation and median. The demographic characteristics of the household members (e.g. age, gender, ethnicity, occupation, marital status, education level, height, weight and health status) will be analysed to examine the influencing factors of the household salt monitoring system by using multivariate regression analysis. Linear mixed models, generalized estimating equations (GEE) or t-test will be used to examine the predictors of added salt intake of each household member and salt consumption-related KAP of the household’s main cook and the moderators, mediators of the intervention effects. Correlation analysis will be used to test the link between the score of salt consumption-related KAP and daily added salt intake of each household member. Meanwhile, we will analyse the time trends of the daily added salt intake of each household member during the study period. The level of significance will be set at < 0.05. For the qualitative data analysis, the audio recordings will be transcribed verbatim by trained transcribers. Content analysis of the qualitative information gathered will be done using MAXqda. In addition, if there is enough data acquired about blood pressure, analysis of the impact of the system on blood pressure will be considered.

## Discussion

This study will be the first to apply IOT technology to monitor and control the added salt intake in Chinese households. We expect to find that the salt consumption-related KAP score will be higher and the daily added salt intake of each household member will be less in the intervention group than in the control group. In the intervention group, we also expect that the time trend of the daily added salt intake of each household member will show an overall downward trend. A stronger reduction is expected in salt over-consumption households, and the strength of the effect is expected to be higher between 3 months and baseline than between 6 months and baseline and between 6 and 3 months due to time effect [16]. We speculate that if the monitoring system is used continuously after 6 months, the salt consumption-related KAP of the household’s main cook will improve continuously or be maintained at a good level, and the daily added salt intake of each household member will decrease continuously or be maintained under the recommended level of the Chinese Nutrition Society (6 g/d) [3]. In addition, the demographic characteristics may be factors that influence the SALTCHECKER effect.

The four main advantages of this study are as follows. First, compared with similar trials aimed at reducing salt intake with simpler approaches [44–46], the SALTCHECKER in the present study could monitor the daily added salt consumption of each household member with less labour cost and more convenience, and reflect on the household’s main cook’s habit of using salt in the real-world setting. Besides, the salt-limiting WeChat programme could provide personalized suggestions to users, which may produce a better salt-limiting effect. Second, a traditional weighing method is considered to be too complicated for accurate assessment of the added salt intake of each household member, although the SALTCHECKER is less accurate, it is simple enough that residents can use by themselves [47]. Third, the system can monitor the household added salt consumption for a long time, which can reflect the habitual added salt intake of household members and reduce measurement error, compared with traditional survey methods. Fourth, as the number of smartphone users worldwide today surpasses three billion and is forecast to further grow by several hundred million in the next few years [48]. China, India, and the United States are the countries with the highest number of smartphone users, with each country easily surpassing the 100 million users [48]. Therefore, we consider a large number of smartphone users could represent a study population and the system could apply to other populous smartphone-using countries.

There are also four main limiting factors in this study. First, the method of estimating the added salt intake of each household member may not be accurate. Although we will take measures to reduce the salt consumption measurement error, we cannot ensure the salt used for cooking or at the table is taken from the salt checker container only. In addition, the salt intake may be from at-home and out-of-home sources, and the average prevalence of food away from home consumption increased from 9.40% to 13.95% between 2004 and 2011 in China [49]. Although at present, the SALTCHECKER can only measure the added salt consumption at home, we will use the salt-limiting WeChat mini programme to obtain information on participants’ dietary behaviours, such as the frequency of eating out, the places to eat, and the number of meals per day. We also hope to have a smarter and more accurate way to monitor salt intake from other sources such as out-of-home eating, ultra-processed foods and condiments containing sodium in the future. Second, we cannot ensure that all household main cooks will persist in receiving the intervention on the salt-limiting WeChat mini programme, which may hinder the intervention effects. To address this limitation, we will send prompting text messages to the participating household’s main cook at a fixed time weekly and set a daily punch-in reminder function in the WeChat mini programme. Besides, we will provide some reward (e.g. a salt spoon with a scale, a sphygmomanometer and a smart watch) to promote adherence to the daily use of the system for the participants. All relevant data can be entered into the registry database in the WeChat mini programme flexibly and dynamically, and which the participants can view their data at any time with the Internet. Third, the salt-limiting effects may be insignificant because the planned intervention time is 6 months. Although we found that 6 months of salt-limiting intervention could have an effect in a previous study [45], we hope the intervention time will be extended and the long-term salt-limiting effects will be reflected in further research. Fourth, this study will be conducted in Chongqing, China, where the dietary habits are different from those in other places. This setting may limit generalisability, but the feasibility of the findings will inform the development of a larger effectiveness trial.

This study will provide a monitoring system for the calculation of added salt intake of each household member and guidance for salt-limiting behaviour, which can be used as a reference for similar studies elsewhere. Through the assessment of the effectiveness of SALTCHECKER, the reduction in the added salt intake of each household member and the improvement in the salt consumption-related KAP of the household’s main cook will provide an excellent way to manage and control salt intake. The system can also be easily incorporated into health management and promotion interventions.

## Trial status

The project is planned from January 2019 until December 2020. The recruitment phase of this study is scheduled from January 2020 to March 2020. The intelligent household added salt monitoring system will be developed and improved from January 2019 until December 2019. The daily added salt intake of each household member will be monitored from April to September and the salt consumption-related KAP of the household main cook will be investigated in April, July and October 2020. Data analysis and evaluation will be performed after September 2020. The protocol stands at version 3.0 on 10 March 2020.

## Supplementary information

**Additional file 1.** SPIRIT 2013 checklist. Recommended items to address in a clinical trial protocol and related documents.

## Data Availability

The data sets used and/or analysed during the current study will be available from the corresponding author on reasonable request.
